# Dr. Binns's Observations on Vision

**Published:** 1805-08-01

**Authors:** Jonathan Binns

**Affiliations:** Lancaster


### *To the Editors of the Medical and Physical Journal.*

GENTLEMEN,

ON reading Dr. Lang's "Observations on the Management and Preservation of the Eyes," in your 77th No. p. 11, I was reminded of the case of a gentleman, who a few years years ago became blind of one eye from some obscure cause. On covering that eye only, and suddenly opening it, no contraction of the pupil was discoverable, as would be supposed: but on covering both eyes, and suddenly exposing them to the light at the same instant, there was not only the usual contraction in the perfect eye, but a manifest contraction in the blind one; which will, perhaps, be allowed to be an additional confirmation of Dr. L's position, that there exist a "sympathy and close connexion of our organs of sight, &c." But the management he recommends for the preservation of the eyes, not coinciding with my experience, I am induced to lay it before you, and, if approved, ye are at liberty to insert it in your publication.

Many years ago my eyes became very tender, probably in consequence of much reading; so that I could not bear to read or write long together without some contrivance, especially if the paper were very white. In this state I found, if I placed the book, or paper, between me and the window, or candle, it was less tolerable than when the light came sideways, or partly from behind: this I account for from the light's being more directly reflected from the paper into the eyes when coming in the first direction; and though my sight of late has improved, I still find the front light less pleasant; and presume that most persons, who are much affected by a strong light reflected from white surfaces, will be very sensible of the difference, and that the present mode of sitting with the left side to the light in writing will still be found most advantageous; for thus the eyes will receive less reflection from the paper than even when the light comes from the right. I own with Dr. L. that in a side light, more rays generally fall on one eye than on the other; but this, if painful, is easily guarded against by a shade, as the hand placed on that side of the face, the elbow resting on the desk, or a candle-screen; for this purpose I prefer the green silk, called Persian to oiled paper, that colour being very friendly to the eyes: yet I found spectacles of green glass not answer the purpose, chiefly from their causing, when removed or not looking through them, every object to appear of a reddish hue.

Being disappointed of getting green plate glass, I had recourse, for relief from the whiteness of paper, to pieces of thin horn, such as are used for lantern lights, and held them upon the book in reading, which I found of some service. I afterwards got the horn stained, by a silk-dyer of a beautiful light green, which answered extremely tremely well. On using this, I was still more sensible of the preference due to a side light; for when placed between me and the light, the reflection from it was very troublesome. I found *that* horn answer best for staining, which is the most free from colour. When my eyes became so strong as not to be in much need of it, I sometimes laid it on the part of the page where I was not reading, or on the other page.

But as blank paper reflects more light than letter-press, the tender sighted, who write much, will perhaps be equally desirous of knowing how they may obtain relief. At first, on using thin writing paper, I put black or green paper under it; but this not answering well, and still worse when the writing paper was thick, recourse was had to another device. A piece of green cloth was pasted on a thin board, 12 inches by 8, in the middle of which an opening was cut 8 inches long and 1 broad, so as to allow of writing two lines; then, by drawing down the board about half an inch, one line remains in sight to direct the penman in writing the next at a proper distance, and so on to the bottom of the page; thus, instead of the eyes being exposed to 60 or 100 inches of white reflecting surface, it is exposed to only 8 inches; and this may be further reduced if the writer will be at the trouble of making use of ruled or black lines. The green cloth, in order to completely cover half a sheet of fool's-cap paper when writing at the top and at the bottom, must necessarily extend 8 or 9 inches above, as well as below, the board. That part of the board, which must slide over the newly written part, should be hollowed out underneath, to prevent the writing from being blotted; or that part of the board, namely, from the opening to the top, may be entirely cut away, and a cross piece laid over, and the cloth over the whole. The upper edge, at least of the lower side of the opening, ought to be pared very thin, so that the pen may not go against it in writing.

This hasty sketch, for relieving my medical brethren and other literary characters that are afflicted with great sensibility to light, will probably be considered as trifling by them that have strong eyes; but I flatter myself it will prove useful to the former, should they be induced to put it to the test.

*Lancaster,*

*13th of 7th Month,* 1805.

JONATHAN BINNS.

OBSERVATIONS

